# Protocol for Establishing National Guidance for Idiopathic Granulomatous Mastitis (ENIGMA)

**DOI:** 10.1093/bjsopen/zraf141

**Published:** 2026-01-19

**Authors:** Shaneel Shah, Leah Argus, Goonj Johri, Daniel Ahari, Rute Castelhano, Sofia Christoforidis, Christopher Darlow, Iain Lyburn, Nisha Sharma, Vijay Sharma, Rudresh Shukla, Kavita Sethi, Emma MacInnes, Roisin Bradley, Claudia Harding-Mackean, Karina Cox, Nazina Arafin, Cliona C Kirwan, North West Breast Research Collaborative, Shaneel Shah, Leah Argus, Daniel Ahari, Rute Castelhano, Sofia Christoforidis, Cliona C Kirwan

**Affiliations:** The Nightingale Centre (Manchester University NHS Foundation Trust), Wythenshawe Hospital, University of Manchester, Manchester, UK; The Nightingale Centre (Manchester University NHS Foundation Trust), Wythenshawe Hospital, University of Manchester, Manchester, UK; Princess Anne Hospital, University Hospital Southampton NHS Foundation Trust, Southampton, UK; The Nightingale Centre (Manchester University NHS Foundation Trust), Wythenshawe Hospital, University of Manchester, Manchester, UK; The Nightingale Centre (Manchester University NHS Foundation Trust), Wythenshawe Hospital, University of Manchester, Manchester, UK; The Nightingale Centre (Manchester University NHS Foundation Trust), Wythenshawe Hospital, University of Manchester, Manchester, UK; Infectious Diseases and Microbiology, University of Liverpool, Liverpool, UK; Infectious Diseases and Microbiology, Liverpool University Hospitals NHS Foundation Trust, Liverpool, UK; Department of Radiology, Gloucestershire Hospitals NHS Foundation Trust, Cheltenham, UK; Cobalt Medical Charity, Cheltenham, UK; Cranfield University, Wiltshire, UK; Department of Surgery and Department Radiology, Leeds Teaching Hospital NHS Trust, Leeds, UK; Infectious Diseases and Microbiology, University of Liverpool, Liverpool, UK; Infectious Diseases and Microbiology, Liverpool University Hospitals NHS Foundation Trust, Liverpool, UK; NIHR Manchester Biomedical Research Centre, Manchester University NHS Foundation Trust, University of Manchester, Manchester, UK; Department of Surgery and Department Radiology, Leeds Teaching Hospital NHS Trust, Leeds, UK; Department of Surgery and Department Radiology, Leeds Teaching Hospital NHS Trust, Leeds, UK; Department of Radiology, York and Scarborough NHS Foundation Trust, York, UK; Department of Breast Surgery, Countess of Chester Hospital NHS Foundation Trust, Chester, UK; Department of Breast Surgery, Maidstone and Tunbridge Wells NHS Trust, Maidstone, UK; The Nightingale Centre (Manchester University NHS Foundation Trust), Wythenshawe Hospital, University of Manchester, Manchester, UK; The Nightingale Centre (Manchester University NHS Foundation Trust), Wythenshawe Hospital, University of Manchester, Manchester, UK; Division of Cancer Sciences, School of Medical Sciences, Faculty of Biology, Medicine and Health, University of Manchester, Manchester, UK

Idiopathic granulomatous mastitis (IGM) is a rare, benign, chronic inflammatory disease of the breast. Evidence around the diagnosis and management of IGM remains limited and inconsistent, particularly in Western countries. Most published studies are case series or retrospective cohorts, with few prospective investigations. In the UK, only four papers involving 29 patients have been published^1^. This raises concerns about under-reporting, under-diagnosis, or differences in healthcare pathways compared with regions such as Asia, and the Middle East, where the incidence of IGM appears higher.

IGM typically presents as a discoloured, painful, palpable breast mass, with a long and often frustrating clinical course^2^. Misdiagnosis as a breast abscess is common, delaying diagnosis. The pathophysiology of IGM remains unclear, with theorized aetiologies ranging from infectious, autoimmune, and hormonal to traumatic causes^3^. Overlap with cystic neutrophilic granulomatous mastitis, which is associated with coryneform bacteria, further complicates the diagnosis of IGM^4^.

Treatment options for IGM are diverse, ranging from conservative management and antibiotics to corticosteroids, immunosuppressants, and surgery; however, none are supported by high-quality evidence^5^. Consequently, patients may endure prolonged and potentially harmful interventions with uncertain benefit.

To address these gaps, the Establishing National Guidance for Idiopathic Granulomatous Mastitis (ENIGMA) collaboration was established, supported by the Association of Breast Surgeons, British Society of Breast Radiology, Royal College of Pathologists, and British Infection Association. ENIGMA is a multidisciplinary, mixed-methods programme (*Fig. 1*) that aims to: evaluate the relevance of current international evidence through a scoping review; understand current UK clinical practice; identify risk factors and diagnostic modalities; appraise treatment options; and achieve expert consensus^6^. Ultimately, ENIGMA seeks to create national guidelines to standardize care, optimize diagnostic pathways, reduce ineffective treatments, and improve patient outcomes, with potential relevance internationally. A comprehensive protocol for ENIGMA is provided in the *Supplementary material*.

**Fig. 1:**
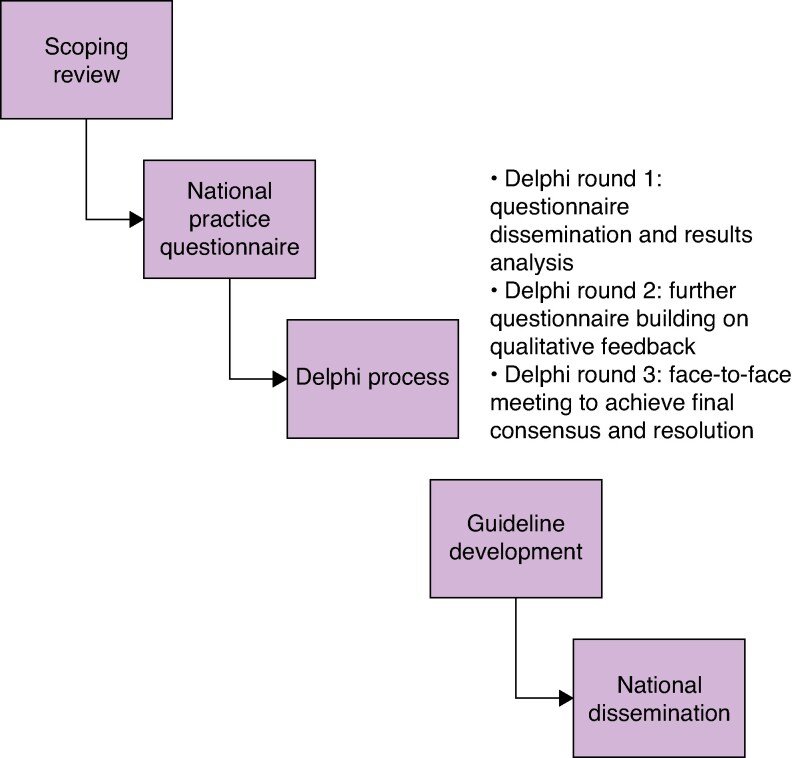
Schematic diagram of the ENIGMA protocol The ENIGMA protocol is a multidisciplinary, mixed-methods programme of work to create guidelines that underpin the diagnosis and management of idiopathic granulomatous mastitis in the UK. ENIGMA, Establishing National Guidance for Idiopathic Granulomatous Mastitis.

The scoping review, registered with PROSPERO (CRD42023397427), will map existing literature across the MEDLINE, Embase, and Cochrane databases. The review will focus on the incidence, presentation, risk factors, diagnostic strategies, treatment modalities, and outcomes of IGM. Case reports, cohort studies, trials, systematic reviews, and meta-analyses will be included.

A nationwide survey will assess how UK clinicians currently diagnose and manage IGM. Separate versions will be tailored for the relevant different specialties (surgery, microbiology and infectious diseases, and rheumatology). The questionnaire will be distributed via national professional bodies and multidisciplinary team coordinators, capturing both unit-level and individual clinician perspectives. The survey will provide valuable context, highlight novel approaches, and help identify experts for the next stage.

A modified Delphi approach will establish expert consensus where evidence is lacking. Multidisciplinary panellists will anonymously evaluate statements on diagnosis, treatment, follow-up, and outcomes across multiple rounds. Iterations will include virtual surveys and a face-to-face meeting to reach agreement thresholds and resolve outstanding issues. Patient members will contribute lived-experience perspectives, especially on treatment acceptability and follow-up strategies. The resulting consensus statements will underpin national guidelines.

Once approved, guidelines will be disseminated through professional associations and multidisciplinary team networks to maximize adoption. The goal is to promote consistent, evidence-informed care pathways across the UK and similar healthcare systems. Longer-term ambitions include collaboration with European centres such as EUBREAST and the Granulomatous Mastitis Registry (GraMaReg) study. Collection of prospective data would support continuous improvement, help refine treatment algorithms, and reduce financial and clinical burdens for both patients and the National Health Service.

IGM is a rare but challenging condition with uncertain aetiology and highly variable management. The lack of consensus risks misdiagnosis, delays, and patient harm. ENIGMA represents the first coordinated UK effort to systematically review evidence, survey national practice, and develop guidelines. By establishing robust diagnostic and treatment pathways, ENIGMA aims to improve patient care, reduce unnecessary interventions, and provide a framework for future research and audit.

## Collaborators

ENIGMA is supported by the North West Breast Research Collaborative (NWBRC), an organization of trainee breast surgeons based in the North West of England. Members of the NWBRC that sit on the ENIGMA steering committee are: Shaneel Shah (The Nightingale Centre, UK), Leah Argus (The Nightingale Centre, UK), Daniel Ahari (The Nightingale Centre, UK), Rute Castelhano (The Nightingale Centre, UK), Sofia Christoforidis (The Nightingale Centre, UK) and Cliona C Kirwan (The Nightingale Centre, UK).

## Supplementary Material

zraf141_Supplementary_Data

## Data Availability

No original data presented in this letter or *Supplementary material*. All data from subsequent research will be shared and made freely accessible.
