# Supplementary material for: Protocol for Establishing National Guidance for Idiopathic Granulomatous Mastitis (ENIGMA)
Source: BJS Open. 2026 Jan 19;10(1):zraf141. doi: 10.1093/bjsopen/zraf141 (PMC12814874; doi:10.1093/bjsopen/zraf141)
Supplement: zraf141_Supplementary_Data [file zraf141_supplementary_data.docx]

# Appendix

Table 1: Search strategy for Ovid MEDLINE(R) electronic database

| Search Number | Search Terms |
| --- | --- |
| 1 | granulomatous mastitis/ |
| 2 | (granuloma* adj4 (mastitis or mastodynia or mastalgia)).ti,ab,kw,kf. |
| 3 | ((lobular or lobar) adj4 (mastitis or mastodynia or mastalgia)).ti,ab,kw,kf. |
| 4 | (cystic neutrophilic adj4 (mastitis or mastodynia or mastalgia)).ti,ab,kw,kf. |
| 5 | ((nonpuerperal or "non-puerperal" or nonlactation* or "non-lactation*" or periductal) adj4 (mastitis or mastodynia or mastalgia)).ti,ab,kw,kf. |
| 6 | 1 or 2 or 3 or 4 or 5 |
| 7 | breast/ or mammary glands, human/ or nipples/ or (breast* or nipple* or areola* or "milk duct*" or "milk gland*").ti,ab,kw,kf. |
| 8 | granulomatous mastitis/ or granuloma*.ti,ab,kw,kf. |
| 9 | 7 and 8 |
| 10 | 6 or 9 |
| 11 | limit 10 to last 20 years |
| 12 | limit 11 to english language |

Table 2: Search strategy for Embase electronic database

| Search Number | Search Terms |
| --- | --- |
| 1 | granulomatous mastitis/ |
| 2 | (granuloma* adj4 (mastitis or mastodynia or mastalgia)).ti,ab,kw,kf. |
| 3 | ((lobular or lobar) adj4 (mastitis or mastodynia or mastalgia)).ti,ab,kw,kf. |
| 4 | (cystic neutrophilic adj4 (mastitis or mastodynia or mastalgia)).ti,ab,kw,kf. |
| 5 | ((nonpuerperal or "non-puerperal" or nonlactation* or "non-lactation*" or periductal) adj4 (mastitis or mastodynia or mastalgia)).ti,ab,kw,kf. |
| 6 | 1 or 2 or 3 or 4 or 5 |
| 7 | exp breast/ or breast abscess/ or (breast* or nipple* or areola* or "milk duct*" or "milk gland*").ti,ab,kw,kf. |
| 8 | granulomatous mastitis/ or granuloma*.ti,ab,kw,kf. |
| 9 | 7 and 8 |
| 10 | 6 or 9 |
| 11 | limit 10 to last 20 years |
| 12 | limit 11 to english language |

Table 3: Search strategy for Cochrane Central Register of Controlled Trials (CENTRAL) and Cochrane Database of Systematic Reviews (CDSR) electronic database

| Search Number | Search Terms |
| --- | --- |
| 1 | MeSH descriptor: [Granulomatous Mastitis] explode all trees |
| 2 | ((granuloma*) NEAR/4 (mastitis or mastodynia or mastalgia)):ti,ab,kw |
| 3 | ((lobular or lobar) NEAR/4 (mastitis or mastodynia or mastalgia)):ti,ab,kw |
| 4 | ((cystic neutrophilic) NEAR/4 (mastitis or mastodynia or mastalgia)):ti,ab,kw |
| 5 | ((nonpuerperal or "non-puerperal" or nonlactation* or "non-lactation*" or periductal) NEAR/4 (mastitis or mastodynia or mastalgia)):ti,ab,kw |
| 6 | #1 OR #2 OR #3 OR #4 OR #5 |
| 7 | MeSH descriptor: [Breast] explode all trees |
| 8 | ((breast* or nipple* or areola* or "milk duct*" or "milk gland*")):ti,ab,kw |
| 9 | #7 OR #8 |
| 10 | MeSH descriptor: [Granulomatous Mastitis] explode all trees |
| 11 | (granuloma*):ti,ab,kw |
| 12 | #10 OR #11 |
| 13 | #9 AND #12 |
| 14 | #13 OR #6 |
| 15 | #13 OR #6 with Cochrane Library publication date Between Jan 2003 and Dec 2023 |

Table 4: Summary of Scoping Review Search

| Category | Item(s) |
| --- | --- |
| Search title | Scoping review of idiopathic granulomatous mastitis: Clinical management and research methodology |
| Population(s) | All patients, all age groups  All stages of management - clinical suspicion, diagnosis, management, follow-up |
| Intervention(s) | All interventions including: surveillance, medical management, surgical management |
| Outcomes | Incidence and prevalence  Diagnostic criteria  Management |
| Exclusion criteria | Not in the English language  Animal studies  In vitro studies  Cadaveric studies  Conference abstracts  Full-text not available  Non-original research (e.g., response/comment, letter to the editor, commentary, literature/narrative/scoping review, opinion piece)  Trial or study protocol  Non-idiopathic granulomatous mastitis (i.e., tuberculosis, sarcoidosis, IgG4-related disease) – with the exception of cystic neutrophilic granulomatous mastitis  Intervention not licensed by Medicines and Healthcare Products Regulatory Authority (MHRA) (e.g., Ayuvredic or Traditional Chinese Medicine therapies) |
| Search period | 2003 - 2023 |
| Comments | Initial sift (study design only) conducted by Information Specialist |

Table 5: Variables included in Data Extraction Proforma

| Category | Variable |
| --- | --- |
| Methodological data | Year of publication  Authors  Country  Site(s) of data collection  Year(s) of data collection  Prospective or retrospective data collection  Study design  Possible risk factor(s)  Study outcome(s)  Sample size  Data missingness  Method of handling missing data |
| Demographic data | Age  Gender  Ethnicity  BMI  Comorbidities  Pregnancy history  Breastfeeding history  Risk factor(s) |
| Investigations | Symptom(s)  Duration of symptomms  Imaging type(s)  Imaging finding(s)  Blood test(s)  Microbiology specimen  Microbiology investigations  Specimen type for histopathology  Histopathological features  Excluded differential diagnoses  Specialties named in patient care |
| Treatment | Observation (including definition, duration, cross-over to other treatment)  Medical (including intervention, dose, route, frequency, duration)  Procedural or surgical (including operation/procedure and timing)  Lines of treatment  Durations between lines of treatment |
| Outcomes | Follow-up duration  Resolution  Definition of resolution  Time to resolution  Recurrence  Time to recurrence  Mortality  Cosmetic surgery following treatment |
